# Targeting Farnesoid X receptor (FXR) for developing novel therapeutics against cancer

**DOI:** 10.1186/s43556-021-00035-2

**Published:** 2021-07-10

**Authors:** Sosmitha Girisa, Sahu Henamayee, Dey Parama, Varsha Rana, Uma Dutta, Ajaikumar B. Kunnumakkara

**Affiliations:** 1 Department of Biosciences and Bioengineering, Cancer Biology Laboratory and DBT-AIST International Center for Translational and Environmental Research (DAICENTER), Indian Institute of Technology Guwahati, Guwahati, Assam 781039 India; 2 Cell and Molecular Biology Lab, Department of Zoology, Cotton University, Guwahati, Assam 781001 India

**Keywords:** Cancer, FXR, Cell signalling, Agonist, Antagonist

## Abstract

Cancer is one of the lethal diseases that arise due to the molecular alterations in the cell. One of those alterations associated with cancer corresponds to differential expression of Farnesoid X receptor (FXR), a nuclear receptor regulating bile, cholesterol homeostasis, lipid, and glucose metabolism. FXR is known to regulate several diseases, including cancer and cardiovascular diseases, the two highly reported causes of mortality globally. Recent studies have shown the association of FXR overexpression with cancer development and progression in different types of cancers of breast, lung, pancreas, and oesophagus. It has also been associated with tissue-specific and cell-specific roles in various cancers. It has been shown to modulate several cell-signalling pathways such as EGFR/ERK, NF-κB, p38/MAPK, PI3K/AKT, Wnt/β-catenin, and JAK/STAT along with their targets such as caspases, MMPs, cyclins; tumour suppressor proteins like p53, C/EBPβ, and p-Rb; various cytokines; EMT markers; and many more. Therefore, FXR has high potential as novel biomarkers for the diagnosis, prognosis, and therapy of cancer. Thus, the present review focuses on the diverse role of FXR in different cancers and its agonists and antagonists.

## Introduction

Cancer has become one of the major universal challenges due to its rising burden and mortality [1–14]. According to the report of GLOBOCAN 2018, an estimate of 18.1 million new cases and 9.6 million deaths occurred annually due to this disease worldwide [15, 16]. Therefore, developing novel targets and drugs has become imperative for the better management of this disease. Modification of various genes and proteins enables normal cells to attain oncogenic behaviours and modulate molecular pathways that lead to cancer development and progression [17–19]. Thus, the increase in alterations leads to changed behaviour of the normal cell systems that otherwise might perform anti-proliferative or tumour suppressive behaviour [20]. Nuclear receptors, a class of proteins found within the cell, are associated with multiple pathways and form an important target for treating several diseases, including cancer [21]. Farnesoid X receptor (FXR) protein, also known as bile acid receptor (BAR), is one such nuclear receptor linked with various cancers. For instance, in breast, lung, oesophagal, and pancreatic cancers, overexpression of FXR is linked with increased proliferation of cancer cells [22]. Again, the activation of FXR was also reported to increase epithelial-mesenchymal transition (EMT) in hepatocellular carcinoma (HCC) through the modulation of EMT markers [23]. As per the cBioPortal database, different mutations in FXR have been reported for various cancers. The mutations mostly include missense, followed by nonsense, frameshift deletion/insertion, and splice-site mutations. In this portal, a total of 10,953 patients and 10,967 samples were analyzed, and results showed 172 somatic mutations with a frequency of 1.3% [www.cbioportal.org].

The FXR protein was first cloned and named as farnesoid X receptor in the year 1995, sharing the sub-class with the metabolic receptors of vitamin D, androstane, pregnane X, and liver X (α and β) [21, 24]. It was formerly named due to its plausible interaction with farnesol [24–27]. Subsequent studies reported bile acids like chenodeoxycholic acid (CDCA) as its primary agonists [25, 27–33]. The normal function of FXR is mostly regulated through its binding to retinoid X receptor (RXR) either in the form of monomer or heterodimer [21]. The RXR binding-regulated function represents its most common form and an example of simple transactivation [34]. The composite transactivation process describes the modulation of FXR by the receptor-like liver receptor homolog-1(LRH-1) and induction of synergistic behaviour by increasing the activity of FXR. Another form of transactivation is the monomeric transactivation, where FXR binds and activates the UDP glucuronosyltransferase 2 family, polypeptide B4 (UGT2B4), in the form of monomer in an RXR-independent manner [34]. Furthermore, FXR regulates the function of glucose transporter type 4 (GLUT4) protein without interfering with the insulin-induced GLUT4 process [35].

FXR is usually expressed in the liver, intestine, kidney, and adrenal glands, where mainly the intestinal and hepatic FXR signalling maintains the inhibited regulation of bile conversion from cholesterol by regulating its rate-limiting enzyme cholesterol 7-alpha-hydroxylase (CYP7A1) [28, 36]. Further, the low expression of FXR was also reported in adipose, breast, and heart tissues [37]. Besides, FXR is involved in the regulation of lipid metabolism by targeting the phospholipid transfer protein (PLTP) and apolipoproteins apoA-I, apoC-II, and apoC-III as evidenced in an in vivo study with a mouse model [38]. The activated FXR also suppressed sterol regulatory element-binding protein 1C (SREBP-1C)-induced lipogenesis [39]. The activation of FXR induces the expression of a small heterodimer partner (SHP) that leads to inhibition of liver X receptor (LXR) and other factors that cause SREBP-1C expression [40]. In addition to its normal function, the high expression of FXR was found to positively regulate the cancer cell proliferation and tumour growth, which might involve the activation of several oncogenes like cyclin D1 in non-small cell lung cancer (NSCLC) [41]. In contrast, FXR also acts as a tumour suppressor in intestinal tumours, and its low expression led to increased tumour growth [42]. It suggests the dual role of FXR as a proto-oncogene or tumour suppressor gene depending upon its tissue function [41]. FXR protein is also involved in regulating various molecules such as tumour necrosis factor-α (TNF-α), p21, B-cell CLL/lymphoma 2 (Bcl-2), nuclear factor kappa-B (NF-κB), and other pro-inflammatory cytokines [43]. Thus, the targeted modulation of FXR represents a molecular basis and an alternative strategy in the prevention and treatment of cancer.

## Structure of FXR

**Fig. 1:**
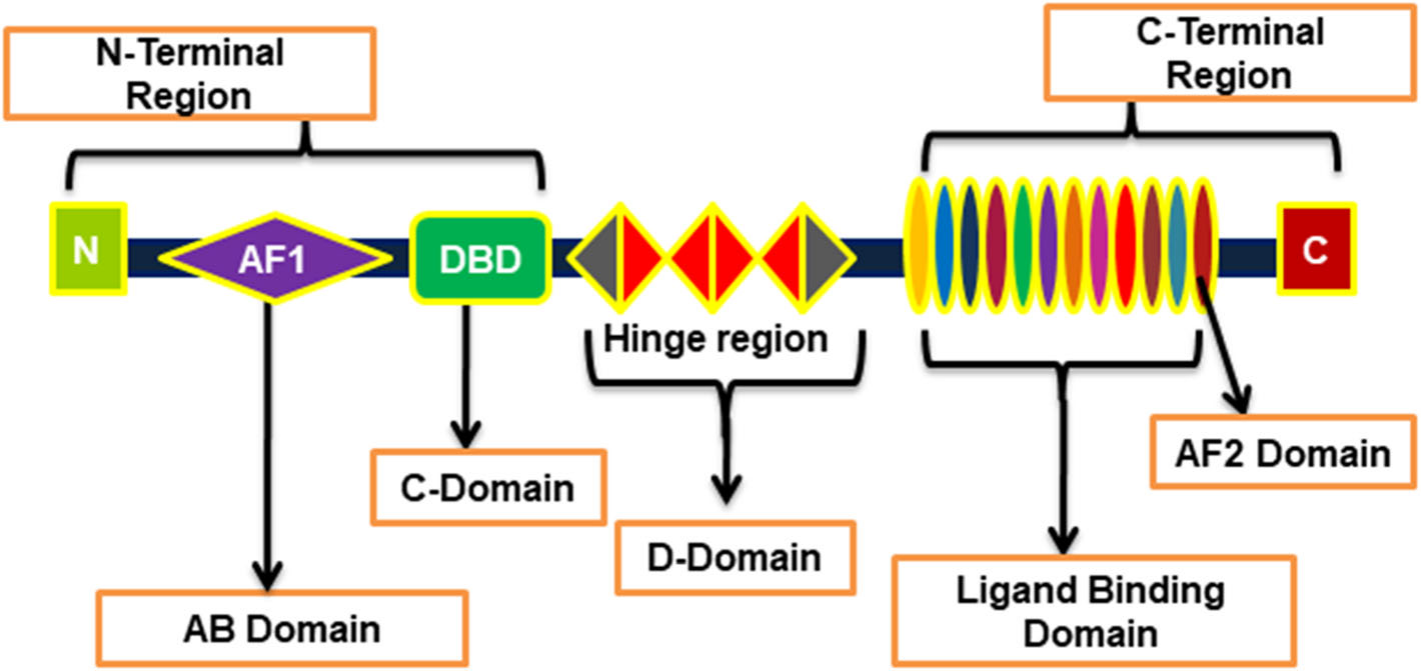
Structure of Farnesoid X receptor (FXR). FXR consists of N-terminal region, hinge region and C-terminal region. The C-terminal region possesses an AF2 domain that plays an important role in activating FXR

The structure of FXR consists of different parts such as the AB domain consisting of activating function (AF)-1 site in the N-terminal region, C-domain that contains a DNA-binding site, D-domain consisting of a hinge region, as well as the ligand-binding E-domain that has an AF-2 activation site in the C-terminal region (Fig. 1). FXR is usually present in the form of FXRα and FXRβ in mammals [21, 44]. The activation of the helix 12 or AF2 domain plays a significant role in activating and regulating the function of FXR [45]. FXR was first cloned in a rat model and was homologous to the RXR-interacting protein 14 (RIP14) [46]. The two forms of FXR, termed RIP14–1 and RIP14–2, were isolated from murine. These two forms vary from each other in their amino terminus as an additional four amino acids are present at the hinge region of RIP14–2 that consists of an extra 12 bp near the DNA binding domain [47]. Further, the FXR in the rat does not contain the extra 12 bp in the hinge region compared to murine RIP14–1. This suggests the importance of amino-terminus or hinge regions representing different isoforms of FXR with different binding and functions [47]. The FXRα gene consists of 11 exons located in the chromosome 12q23.1 in humans [46]. Further, in humans and other rodent species, FXRα is exhibited from a single gene locus that has a different promoter and RNA splicing sites that give rise to its four isoforms, i.e., FXRα1, FXRα2, FXRα3, and FXRα4 [48]. In the case of FXRβ, it is regarded as a pseudogene in humans and primates, while it is functionally expressed in animals like dogs, rabbits, and rodents. The FXRβ is known to be sensitive to lanosterol, a precursor of sterols, although its exact biological role is unknown [49].

In the absence of a ligand, the inactive FXR is bound to the FXR responsive elements (FXREs) of the target genes in the form of a heterodimer along with RXR and other co-repressor proteins [37]. When the bile acids bind to the ligand-binding region of the FXR, it activates FXR and its target gene SHP (*NR0B2*) that can form dimers with liver receptor homolog 1(LRH1) and oxysterol-responsive liver X receptor α (LXRα) [49]. The binding of bile acids to FXR results in a conformational change that causes the release of transcriptional repressor proteins such as nuclear receptor co-repressor 1 (NCoR) as well as recruitment of co-activator-associated arginine methyltransferase 1 (CARM1) and basal transcription to the responsive element and activate the transactivation process [37, 50].

## Functions of FXR

Several functions of FXR have been reported in highlighting its role in bile acid metabolism and transport, lipid metabolism, glucose metabolism, hepatoprotection, xenobiotic detoxification, and anti-bacterial activity [48, 51]. The conversion of cholesterol to bile acids is essential in maintaining bile acid metabolism and eliminating cholesterol from the body [52]. The rate-limiting enzyme CYP7A1 plays a vital role in the classical pathway of bile acid synthesis by initiating 7α-hydroxylation of cholesterol in the liver [53]. CYP7A1 is induced by the transcription factors LRH1 and LXRα [49]. However, the LXRα response element is not conserved in the human CYP7A1 [54]. The bile acids interact with the binding domain of FXR that subsequently activates its target genes, such as SHP, which inhibit CYP7A1 expression by binding to LRH1 and LXRα [49, 55]. FXR also regulates bile acids through the FXR- fibroblast growth factor (FGF)15/19 pathway, where an activated FXR binds to the second intron of the FGF15 [27]. This binding results in the secretion of FGF15, which then bind to fibroblast growth factor receptor (FGFR)4 on the cell surface of hepatocytes that lead to the activation of c-Jun N-terminal kinases (JNK) pathway and inhibition of CYP7A1 and cytochrome P450, family 8, subfamily B, polypeptide 1 (CYP8B1) [49]. FGF15 also initiates the storage of bile in the gall bladder. Further, FGF19, the ortholog of FGF15, also possesses the conserved binding site for FXR in the human, mouse, and zebrafish, where its activation by FXR represses the synthesis of bile acids [27].

FXR is also involved in regulating lipid metabolism [56]. A recent study suggests the role of FXR in decreasing lipid levels by reducing the synthesis of fatty acids and triglycerides via SHP-mediated activation of FXR. FXR also inhibits LXR and its target SREBP-1C associated with the metabolism and regulation of lipids [57]. The activation of FXR also leads to the reduction in de novo lipid synthesis [58]. Further, FXR also exerts a pivotal role in glucose metabolism in type II diabetes patients [59]. This was confirmed from an in vivo study where an FXR deficient mouse developed glucose intolerance and increased insulin resistance, resulting in elevated hepatic triglycerides, cholesterol, and lipid accumulations [60]. Further, the low expression of FXR leads to an altered insulin signalling in the liver, muscles, and adipose tissues due to the increased expression of free fatty acids (FFA) [61]. This limits the suppression of gluconeogenesis and its related genes, increasing the glucose levels, and decreasing its uptake in the muscle tissues that lead to modified glucose metabolism. Altogether, these findings suggest the association between fatty liver disease and insulin resistance in diabetic conditions [38].

Besides, FXR is also involved in the suppression of inflammation by inhibiting the inflammatory factors such as interleukins, NF-κB, TNF-α, and intercellular adhesion molecule (ICAM)-1 in the liver [62, 63]. The activated FXR also initiates anti-inflammatory proteins such as kininogen, leading to suppression of inflammatory disorders like systemic lupus erythematosus and inflammatory bowel disease (IBD) [58, 64]. NF-κB plays a significant role in modulating the genes involved in inflammation [65, 66]. The sumoylation of FXR was reported to protect liver inflammation by suppressing the inflammatory gene products regulated by NF-κB [58].

FXR was also reported to exhibit anti-fibrotic activity in various disease models. The FXR-deficient mice showed an increase in inflammation and fibrosis in the liver [67]. However, the activation of FXR by obeticholic acid (OCA) suppressed inflammation and fibrosis in primary biliary cholangitis (PBC) patients [68]. Further, OCA was reported to improve bleomycin-induced lung fibrosis in a rat model [69]. The FXR agonist, OCA, was reported to inhibit inflammation and fibrosis in cirrhotic models by decreasing the fibrosis-inducing cells i.e., hepatic stellate cells [70]. Similarly, the treatment of OCA in the monocrotaline (MCT)-induced lung hypertension model improved the lung function and reduced the thickening of the vascular wall, and help in balancing the relaxation and contraction of the lungs [71]. Further, the activation of FXR alleviates renal fibrosis by suppressing the expression of Smad3 and fibronectin [72]. An FXR deficient mouse was also reported to show reduced atherosclerotic injury via reduced levels of LDL cholesterol [73]. Besides, FXR plays a crucial role in tumorigenesis as activated FXR was associated with the development of a premalignant state in oesophagus, known as Barett’s oesophagus, by inducing inflammation [74]. In contrast, its decreased or null expression is associated with increased tumor incidence in colon and liver cancers [75, 76]. The activated FXR inhibited cell proliferation and induced cell cycle arrest in colon cancer by targeting the miR-135A1/Cyclin G2 axis [77]. FXR also plays an important role in ameliorating gallstone disease [78]. Studies showed that FXR deficient mice fed with a lithogenic diet are more prone to gallstones and the treatment with FXR agonist, GW4064 attenuates the condition by inducing bile salt export pump (BSEP), multidrug resistance gene (MDR)-2 and by transporting liver bile acids to bile [61].

## Post-transcriptional modifications (PTM) of FXR

PTMs of a protein plays an essential role in controlling its function and signalling [79, 80]. The PTMs of protein such as ubiquitination and phosphorylation regulate the stability, conformational change, and localization of a protein [81]. As FXR is involved in the export of bile salt, the activation of the transport pump requires the histone methylation of FXR by the histone H3-lysine-4 methyltransferase mixed-lineage leukaemia 3 (MLL3) and histone H4 arginine 3 (H4R3) arginine methyltransferase-1. Another modification of FXR consists of methylation at lysine 26 residues by the enzyme histone-lysine N-methyltransferase (HMT) containing conserved SET domain protein 7/9 (SET7/9) that increases the binding efficiency of FXR and RXR complex to FXRE and induce the expression of its target genes [82].

Studies suggest that various metabolites and cofactors, such as nicotinamide adenine dinucleotide (NAD) and acetyl coenzyme A (A-CoA), control the acetylation process of the FXR [83]. Further, the acetylase activity of p300 regulates FXR through acetylation of the target genes and their receptors, which could be further augmented by FXR agonists. However, the inhibition of this activity results in decreased action of FXR and its partner i.e., SHP protein [84]. Again, the acetylation of FXR at lysine 157 and 217 residues enhanced its stability and inhibits the binding efficiency of FXR to RXR, which lead to decreased activity of FXR. This suggests the dual-role of the p300 acetyltransferase [83]. The sirtuin 1 (SIRT1) deacetylase also regulates the acetylation of FXR as the decreased expression of endogenous liver SIRT1, a positive LXR regulator, increases the acetylation of FXR [85]. On the contrary, activation of SIRT1 by natural compounds like resveratrol decreased the acetylation process of FXR [86]. This suggests that p300 and SIRT1 are two conversely regulated enzymes, and the abrogation of this balanced system might associate with metabolic diseases [83, 85, 86].

Phosphorylation also plays an important role that involves the introduction of phosphate groups to the amino acids such as serine, threonine, and tyrosine residues where kinases are known to participate in the process [87]. Studies have reported the ability of protein kinase C (PKC) to phosphorylate FXR at the sites of serine 135 and 154 of its DNA-binding region [88]. PKC, in the presence of agonists or antagonists, induce the interaction of FXR to peroxisome proliferator-activated receptor gamma coactivator 1-alpha (PGC-1α) and increase the activation of FXR [89]. The FXR activated by GW4064 increases its phosphorylation at serine 154 sites in the nucleus in the presence of vaccinia-related kinase 1 (VRK1), possibly through the recruitment of kinase to FXR that regulate its direct phosphorylation [90]. Also, the familial intrahepatic cholestasis 1 (FIC1), which has a vital role in transporting bile acid, increases the phosphorylated activation of FXR [91]. The PKC zeta mediated phosphorylation of FXR at threonine 442 sites in FXR mutants using antagonists or siRNAs confirms the critical step for FIC1 activity and increases FXR activity as well as its accumulation in the nucleus [89]. The inhibition of PKC zeta decreased FIC1 mediated-phosphorylation and nuclear translocation of FXR that might result in liver diseases [92]. Further, adenosine monophosphate-activated protein kinase (AMPK) is reported to phosphorylate FXR at serine 250 residue [93]. Therefore, it is observed that the activation of AMPK causes hindrance in the selective binding of FXR to co-activators and thereby inhibits the transcriptional activity of FXR as well as its target genes [94]. This resulted in less removal of bile acids that led to an increased liver injury in a cholestasis mouse model [83, 93].

Moreover, ubiquitination and sumoylation play a crucial role in the PTMs of protein [95–97]. Ubiquitination is initiated by the addition of ubiquitin molecules that cause the degradation of proteins [98]. Sumoylation occurs through the covalent and reversible binding of a small family of proteins, small ubiquitin-like modifiers (SUMO) to the lysine residues of the targets that regulate cell processes like DNA repair and apoptosis [96]. Sumoylation can also cause the modification of various cell processes like transcription, protein localization, and mitochondrial activity [99]. The proteasome inhibitor, MG132 induces ubiquitination of FXR in HepG2 cells [89]. Further, the sumoylation of FXR by SUMO2 also increases ubiquitination of FXR dose-dependently. The process of ubiquitination can be inhibited by suppressing the SUMO or by using an FXR agonist [100]. Another study suggests the covalent interaction of SUMO1 to lysine 122 and 275 in the AF-1 and ligand-binding domains of FXR that resulted in decreased transcriptional activity of FXR [101]. Further, the overexpression of SUMO1 minimizes the binding or recruitment of FXR and FXR/RXR to the BSEP and SHP promoters, resulting in decreased activation of BSEP and SHP. However, the abolishment of FXR-sumoylation or knock-down of SUMO1 via siRNA reverses the process. The alteration of this process is associated with liver diseases like cholestasis [99]. The sumoylation of FXR at lysine 325 was also reported through a non-classical pathway regulated by casein kinase 2 (CK2) [100]. Additionally, O-GlcNAc transferase caused O-GlcNAcylation of FXR at serine 62 within the AF-1 domain of FXR, leading to high stability and activity of FXR in response to the glucose levels [102].

## Role of FXR in various types of cancers

**Fig. 2:**
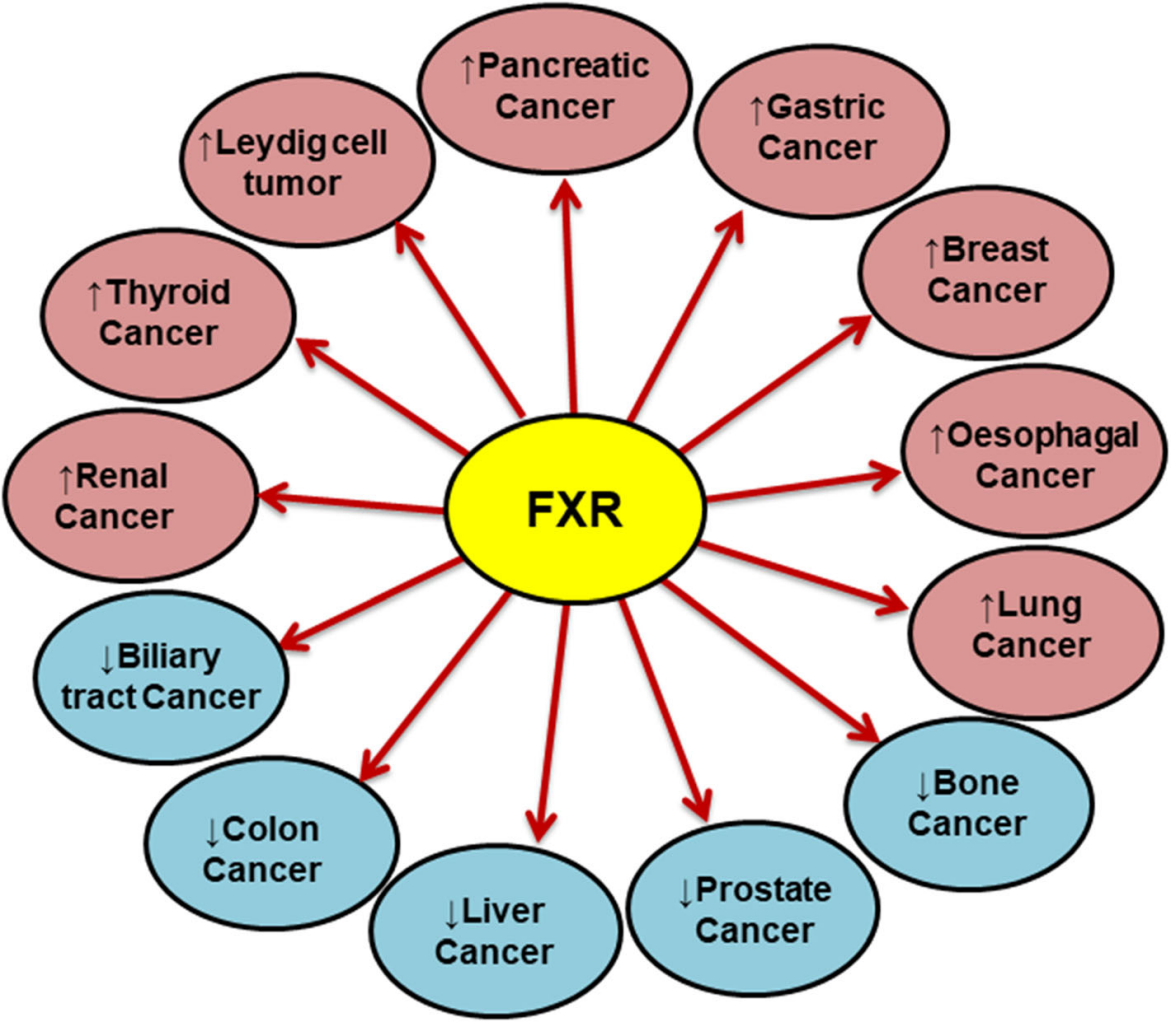
Expression of FXR in various cancers. The arrow in the circle represents the expression of FXR in the respective cancers. “**↑**” represents overexpression of FXR and “↓” represents the low expression of FXR

**Fig. 3:**
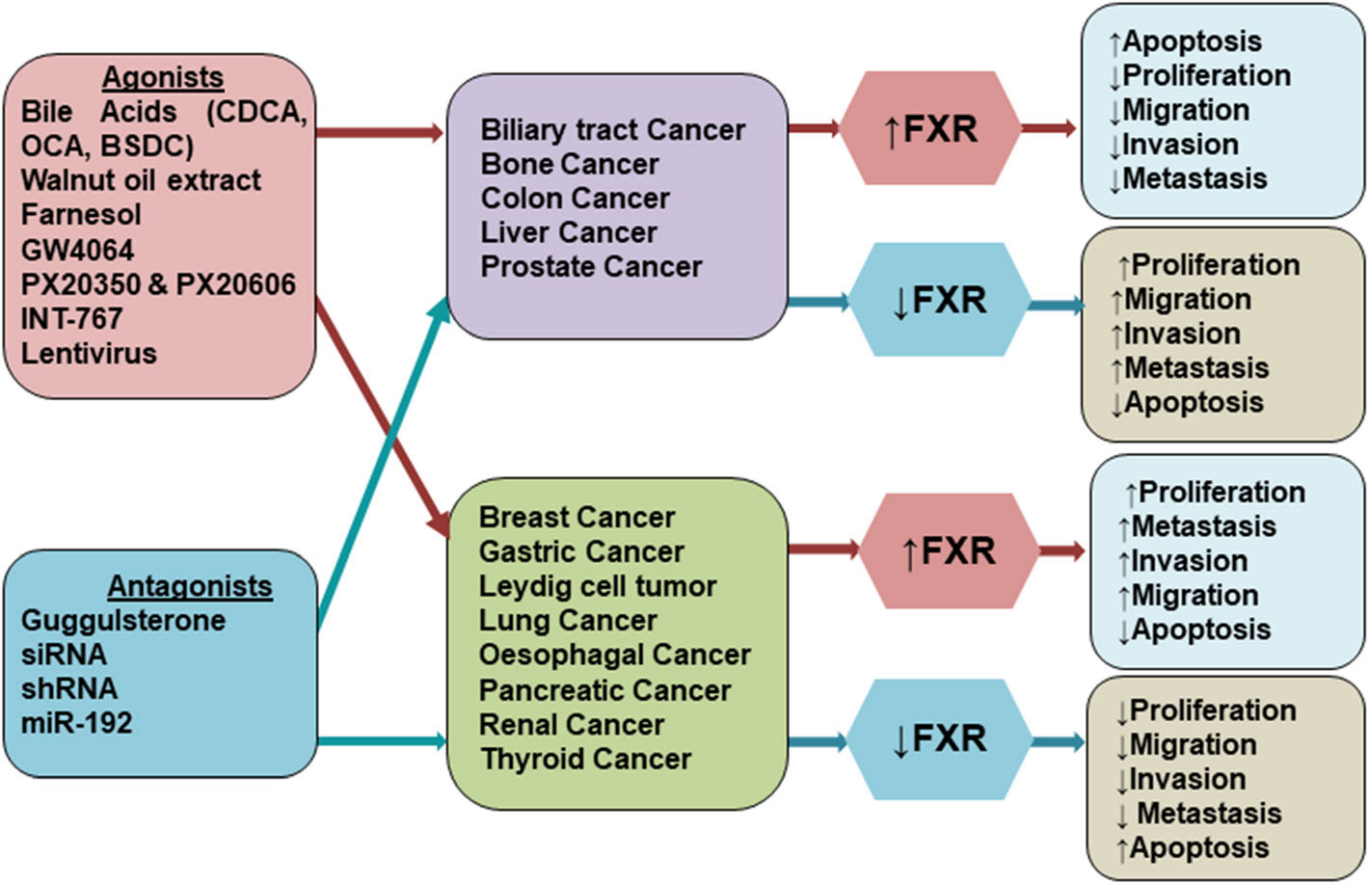
Role of agonists and antagonists in different cancers. The FXR is downregulated as compared to basal level in colon, liver, prostate, bone and biliary tract cancers and the treatment with FXR agonists increase the expression of FXR that leads to inhibition in proliferation, migration, and invasion of cancer cells, and induction of apoptosis. While in other cancers with overexpressed FXR such as breast, gastric, Leydig cell, lung, oesophagal, pancreatic, renal and thyroid cancers, the treatment with agonists increases FXR that results in high proliferation, migration, and invasion, and suppression of apoptosis of cancer cells. Conversely, the treatment with FXR antagonists in FXR-downregulated cancers, it further decreases the expression of FXR that results in induction of proliferation, migration, and invasion, and inhibition of apoptosis. While in FXR-overexpressed cancers, the treatment with antagonists inhibits FXR and decreases the proliferation, migration, and invasion, and induces apoptosis

In addition to its specific role and function in metabolism and diseases, FXR also plays a vital role in cancer. The expression level of FXR varies in different cancers (Fig. 2), and the presence of its agonists/antagonists complement the diversified outcome of FXR in these cancers (Fig. 3). The role of FXR in various types of cancers in the presence of agonists/antagonists is summarized in Table 1. The multiple studies involving the mechanistic role of FXR in different cancers are discussed below.

**Table 1:** Mechanistic role of FXR in various cancers in the presence of its agonists/antagonists

Cancer type	*In vitro/* *In vivo*	Cell Lines/Models	Agonists/Antagonists	Effect/Result	Reference
Bone cancer	In vitro	MG-63	GW4064 (Ac)	↓CCNG1, ↓Bcl-2, ↓cell proliferation, ↑miR-23b-3p, ↑caspase-3, ↑Bax, ↑G1 cell cycle arrest, ↑apoptosis	[103]
			siFXR (In)	↓cleaved miR-23b-3p, ↑CCNG1	[103]
Breast cancer	In vitro	MDA-MB-231	CDCA (Ac)	↑RUNX2, ↑OPN, ↑OC, ↑BSP	[104]
	In vitro	MDA-MB-231	Z-guggulsterone (In) LCA (In)	↓RUNX2, ↓OPN, ↓OC, ↓BSP	[104]
	In vitro	MCF-7	CDCA (Ac)	↑RUNX2, ↑OPN, ↑OC, ↑BSP	[104]
	In vitro	MCF-7	Z-guggulsterone (In), LCA (In)	↓RUNX2, ↓OPN, ↓OC, ↓BSP	[104]
	In vitro	breast CAFs cells	GW4064 (Ac)	↓Cell migration, ↓ILK,↓RhoA-C, ↓Cdc42, ↓Rac1–3, ↓p- Akt, ↓p- MLC	[105]
	In vitro	MCF-7, T47D	GW4064 (Ac)	↓Cell colony	[105]
	In vitro	MCF-7, T47D	Guggulsterone (In)	↑Cell migration	[105]
	In vitro	MCF-7, SKBR3, MDA-MB-231	GW4064 (Ac)	↓ObR mRNA, ↓cyclin D1, ↓survivin, ↑SOCS3	[106]
	In vivo	MCF-7/CAF xenografts	GW4064 (Ac)	↓Tumor growth, ↓Ki67, ↓ Ob, ↓cyclin D1, ↓survivin, ↑ SOCS3,	[106]
	In vitro	MCF-7, MDA-MB-231	CDCA (Ac), GW4064 (Ac)	↑Cyt-c, ↑apoptosis	[107]
	In vitro	FXR-DN MCF-7, MDA-MB-231	CDCA (Ac), GW4064 (Ac)	↓Apoptosis	[107]
	In vitro	MCF-7	Walnut oil extract (Ac)	↓ Cell proliferation	[108]
	In vitro	MCF-7 TR1	CDCA (Ac)	↓HER-2, ↓NF-κB, ↓cell proliferation	[109]
	In vitro	SKBR-3	CDCA (Ac)	↓HER-2, ↓cell colony growth, ↓ancorage-dependent cell growth	[109]
	In vitro	MCF-7	CDCA (Ac)	↓ER, ↑PR	[110]
	In vitro	MCF-7	Farnesol (Ac)	↑PR, ↑cell proliferation	[111]
	In vitro	MCF-7, MDA-MB-468	GW4064 (Ac)	↑Cell death, ↑apoptosis, ↑SHP, ↑IBABP, ↑MRP2, ↓aromatase, ↓GADD 45β, ↓MDR3, ↓MRP1, ↓SLC7A5	[112]
	In vitro	MDA-MB-231	Z-guggulsterone (In)	↑Apoptosis, ↓cell migration	[113]
	In vitro	MDA-MB-231	Z-guggulsterone (In) +BSDC (Ac)	↓Apoptosis	[113]
	In vitro	MDA-MB-231	BSDC (Ac)	↑uPA, ↑uPAR	[113]
BTC	In vitro	GBC-SD, RBE	GW4064 (Ac), CDCA (Ac)^a^	↓Cell viability, ↓Bcl-xL, ↓p-STAT3,↑apoptosis,↑SHP	[114]
	In vivo	GBC-SD cells transplant	GW4064 (Ac)	↓Tumor growth, ↓Bcl-xL, ↓p-STAT3, ↑SHP	[114]
	In vitro	HCCC-9180, GBC-SD, SSP25, RBE cells	miR-421 (In)	↓FXR, ↓BSEP, ↑cell proliferation	[115]
	In vitro	anti-miR-421-induced BTC cells	Farnesol (Ac)	↑FXR, ↑G0/G1 cell cycle arrest	[115]
	In vitro	iCCA primary cells	OCA (Ac)	↓Cell proliferation, ↓colony and spheroid formation, ↓cell migration, ↓Bcl-xL, ↑apoptosis,↑SHP	[116]
	In vitro	iCCA primary cells	CDCA (Ac)	↓Cell proliferation	[116]
	In vivo	iCCA xenograft	OCA (Ac)	↓Tumor growth, ↑necrosis, ↓PCNA+ cells	[116]
	In vitro	EGI1, TFK1	OCA (Ac)	↓Cell proliferation, ↑apoptosis,↓Ki67, ↓PCNA, ↓cyclin D1, ↓cyclin D3	[117]
	In vivo	CD1 nude mice	OCA (Ac)	↓Tumor growth, ↓Ki67,↓ PCNA	[117]
	In vitro	QBC 939	GW4064 (Ac), GS (In)	↓Cell proliferation	[118]
	In vivo	nude mice xenograft	GW4064 (Ac)	↓Tumor growth	[118]
	In vitro	HuCCT	OCA (Ac)	↓IL-6, ↓cell proliferation, ↓migration, ↓invasion, ↓EMT, ↓E-cadherin, ↓ZO-1, ↓β-catenin, ↑N-cadherin, ↑snail, ↑vimentin	[119]
	In vitro	CCLP1, RBE	FXR shRNA (In)	↑IL-6, ↑cell proliferation, ↑migration, ↑invasion, ↑E-cadherin, ↑ZO-1, ↑β-catenin, ↓N-cadherin, ↓snail, ↓vimentin	[119]
	In vivo	OD-SCID mice	OCA (Ac)	↓IL-6, ↓tumor growth, ↓lung metastasis	[119]
Colon cancer	In vitro	HT-29, Caco-2, HCT-116	siFXR (In)	↑Wnt/β-catenin, ↑β-catenin/TCF4	[120]
	In vitro	HT-29, Caco-2, HCT-116	GW4064 (Ac)	↓Cell proliferation	[120]
	In vivo	C57BL/6 mice	–	↓FXR, ↑β-catenin	[120]
	In vitro	HT-29	pCDNA3.1hFXR (Ac)	↓MMP-7, ↓cell proliferation	[121]
	In vitro	HT-29	CDCA, GW4064 (Ac)	↓MMP-7	[121]
	In vitro	HT-29	Guggulsterone (In)	↑MMP-7	[121]
	In vitro	MC38	6E-CDCA (Ac)	↓Cell invasion	[121]
	In vivo	FXR knockout mice (B6.129X1 (FVB)-Nr1h4^tm 1Goz^/J)	–	↑ MMP-7	[121]
	In vitro	SW620, HCT116	GW4064 (Ac)	↓miR-135A1, ↑CCNG2,↑cell death	[77]
	In vitro	SW620, HCT116	FXR siRNA (In)	↑miR-135A1, ↓CCNG2	[77]
	In vitro	HCT-116	APC knockdown	↑ c-Myc, ↓ FXR expression	[122]
	In vivo	APC^min/+^ mice	APC silencing	↓ FXR, ↓ SHP, ↓IBABP,↑COX2	[122]
	In vitro	HCT116	CDCA (Ac) DCA (Ac)	↑miR-22, ↑FGF19, ↓CCNA2 ↑miR-22	[123]
	In vitro	H508, SNU-C4	GW4064 (Ac)	↓Cell proliferation, ↓p-EGFR, ↓p-Src(Tyr416), ↓p-ERK1/2	[124]
	In vitro	HT-29	Guggulsterone (In)	↑Cell proliferation, ↑p-EGFR, ↑p-Src(Tyr416), ↑p-ERK1/2	[124]
	In vitro	SNU-C4	FXR siRNA	↑p-EGFR, ↑p-Src(Tyr416), ↑p-ERK1/2, ↑cell proliferation	[124]
	In vitro	HT-29	pcDNA3.1hFXR	↓Cell proliferation, ↓p-EGFR ↓p-Src(Tyr416), ↓p-ERK1/2	[124]
	In vivo	HT-29 xenograft	–	↓Tumor growth	[124]
	In vitro	Human intestinal mucosa section	–	↓FXR, ↑tumor growth	[125]
	In vivo	FXR^+/-^Apc^Min/+ ^C57BL/6 mice	–	↓Survival, ↑Size and no. of tumors	[126]
	In vitro	Caco-2, HT-29, SW620, SW480	OCA, GW4064(Ac)	↑IBABP mRNA	[127]
	In vitro	HCT116, SW480, DLD1	GW4064	↑DR5	[76]
	In vitro	BGC-823	GW4064 (Ac)	↑CDCA induced CDX2	[128]
			Guggulsterone (In)	↓CDCA induced CDX2	
	In vivo	FXR KO mice	–	↑Cell proliferation, ↑IL-6, ↑cyclin-D1, ↑adenoma	[129]
Esophageal Cancer	In vitro	SKGT-4 TE-3, TE-12, SKGT-4, SKGT-5	FXR shRNA (In) Guggulsterone (In)	↓Cell growth, ↓proliferation ↓cell viability, ↓RAR-β2-led COX-2, ↓MMP-9, ↑apoptosis, ↑Caspase-3, − 8, − 9	[130]
	In vivo	SKGT-4 cells xenograft	FXR shRNA (In), Guggulsterone (In)	↓Tumor size and weight	[130]
	In vitro	TE7	DCA(Ac)	↑IBABP, ↑SHP, ↑MIP3α, ↑IL-8, ↑disease progression, ↓apoptosis ↓	[74]
Gastric Cancer	In vitro	AGS	CDCA(Ac)	↑K13, ↓inflammation-mediated apoptosis	[131]
	In vivo	FXR-KO mice	–	↑K13	[131]
	In vivo	FXR-KO C57BL/6 mice	–	↓Apoptosis, ↓cell damage	[131]
LCT	In vitro	R2C	GW4064(Ac)	↑p53, ↑p21/Cip1, ↑FXR/NF-κB binding	[132]
	In vivo	R2C nude mice xenograft	GW4064(Ac)	↑p53, ↑p21/Cip1, ↑apoptosis, ↓tumor growth	[132]
Liver Cancer	In vivo	C57BL/6 mice	GW4064 (Ac)	↓Gank, ↑p53, ↑C/EBPα, ↑HNF4α, ↓liver cancer development,	[133]
	In vitro	HepG2	OCA (Ac)	↓Gank, ↓cell proliferation,	[133]
	In vitro	SK-Hep-1	GW4064 (Ac)	↑G0/G1 phase arrest, ↓mTOR/S6K, ↓cell proliferation	[134]
	In vitro	Huh-7	FXR siRNA (In)	↓G0/G1 phase arrest, ↑mTOR/S6K, ↓cell proliferation	[134]
	In vitro	HepG2, Huh7	GW4064 (Ac)	↓Cell proliferation, ↑G1 phase arrest, ↑p21, ↓p-STAT3, ↑SOCS3	[135]
	In vivo	HepG2 xenograft	GW4064 (Ac)	↓Tumor growth, ↑SOCS3, ↑p21, ↓p-STAT3	[135]
	In vivo	Crl:NU-Foxn1 nu mice	PX20606 (Ac)	↓Tumor growth, ↓metastasis in lymph nodes	[136]
	In vitro	SK-Hep-1	PX20350 (Ac) PX20606 (Ac)	↓Cell proliferation ↑NDRG2	[136]
	In vitro	HepG2, Huh-7	OCA (Ac)	↓Cell proliferation, ↑cell cycle arrest, ↓invasion, ↓migration, ↓p-STAT3, ↓JAK-2, ↓IL-1β, ↓IL-1β, ↑caspase-3, ↑SHP, ↑SOCS3	[137]
	In vitro	SNU-449	OCA (Ac)	↓Cell proliferation	[137]
	In vitro	HepG2, Huh-7, SNU-449	Guggulsterone (In)	↑Cell proliferation	[137]
	In vivo	FXR/SHP KO Mice	–	↑Gank, ↓C/EBPα	[138]
	In vitro	Hepa 1–6	CDCA, GW4064 (Ac)	↓Gank, ↑C/EBPβ, ↑HDAC1	[138]
	In vivo	C57BL/6 J mice ob−/−Fxr−/−		↑Cdc25b, ↑cyclin D1, ↑FoxM1 ↑liver carcinogenesis	[139]
	In vitro	Human hepatoma Alexander cells, SK-Hep-1, HepG2	FXR + RXR (in presence of GW4064)	Protection against cisplatin toxicity, ↑MOC-1b (ABCB4), ↑MOC-4 (TCEA2), ↑MOC-5b (CCL14, CCL15 and K13)	[140]
	In vitro	Huh7	CDCA (Ac) DCA, LCA, CA (Ac)	↑miR-22, ↑SHP, ↓CCNA2 ↑miR-22	[123]
	In vitro	MIHA cells (with HBx-Δ14 and HBx-Δ35)	Guggulsterone (In)	↓ Hepatospheres, ↓cell migration	[141]
	In vitro	Huh-7, Hep 3B	GW4064, OCA (Ac)	↑Actin polymerization, ↑N-cadherin, ↑SNAI1,↑NR0B2, ↑EMT	[23]
	In vitro	Huh-7, Hep 3B	CDCA (Ac)	↑Actin polymerization,↑N-cadherin, ↑NR0B2, ↑EMT	[23]
	In vitro	Huh-7, Hep 3B	TGF-β	↑Actin polymerization, ↑N-cadherin, ↑EMT, ↓NR0B2	[23]
	In vitro	Huh-7, Hep 3B	Guggulsterone (In)	↓Actin polymerization, ↓N-cadherin, ↓NR0B2, ↓EMT	[23]
	In vitro	HepG2, Huh-7	LV-FXR-GFP (Ac)	↓Cell proliferation, ↑SHP	[142]
	In vivo	HepG2 xenograft	–	↓Tumor growth, ↑SHP	[142]
	In vitro	Hep3B, Huh7,HepG2, PLC, SMMC-7721	GW4064 (Ac)	↑miR-122, ↓IGF-1R, ↓cyclin G1	[143]
	In vitro	Hep3B, Huh-7,HepG2, PLC, SMMC-7721	FXR siRNA (In)	↓miR-122, ↓IGF-1R, ↓cyclin G1	[143]
	In vivo	HCC xenografts	GW4064 (Ac)	↓Tumor growth, ↓IGF-1R, ↓cyclin G1, ↑miR-122,	[143]
	In vivo	C57BL/6 J (FXR−/−/SHPTg)	–	↓Liver malignancy, ↑apoptosis, ↑cyclin D1, ↑p-STAT3, ↑p-JAK-2, ↑IL-6	[144]
	In vivo	C57BL/6 J Fxr−/− FVBN/Abcb4−/−	INT-767 (Ac)	↑Cell necrosis Tumor growth, ↓Cyp7a1, ↓F4/80, ↓IL-1β, ↓IL-6, ↓TNF-α, ↓cyclinD1, ↓PCNA, ↓α-Sma, ↓Col1a1, ↑hepatoprotection, ↑FGF15, ↑SHP	[145]
	In vivo	FXR−/− mice	–	↑Liver injury, ↑TNFα, ↑IL-6,↑IL-1β, ↑Col6a3, ↑Col14a1, ↑MMP-9, ↑TIMP-2, ↑Col6a2, ↑Col5a2, ↑Col3a1, ↑MMP-2, ↑MMP-3, ↑TGFβ1, ↑Sma-α	[146]
	In vitro	WT-HSC FXR−/− HSC	GW4064 (Ac) GW4064 (Ac)	↓PAI-1, ↓Col-α1 No change in PAI-1, Col-α1	[146]
	In vivo	FXR-KO mice	–	↑ Hepatic tumors, ↑Wnt/β-catenin,↑Ser9-phosphorylated, (inactive), GSK-3β, ↑cyclin D1, ↑Dvl, ↑Wnt4, ↓ Ser45-Thr41-phosphorylated, (inactive) β-catenin, ↓ E-cadherin	[147]
	In vitro	human HCC tissues	–	↓FXR, ↓SHP, ↓BSEP	[147]
	In vitro	FXR KO- Huh-7	siRNA (In)	↑β-catenin, ↑cyclin-D1, ↑c-Myc,	[148]
	In vitro	Huh-7 cells	GW4064(Ac)	↓β-Catenin/TCF4 complex,	[148]
	In vitro	HepG2	GW4064(Ac)	↓p-JNK1/2, ↓ROS, ↑SOD3	[51]
	In vivo	FXR−/−C57BL/6-mouse		↑p65, ↓p- ERK, ↑p-JNK1/2	[51]
	In vivo	Mice	PX20606(Ac)	↑HRG	[149]
	In vitro	Huh-7 & HepG2	–	↓BSEP	[150]
	In vitro	HCC tissues	–	↓BSEP, ↑TNF-α, IL-6	[150]
	In vitro	HepG2	siRNA (In)	↑p16/INK4a, ↓HNF-4α, ↓cell proliferation	[151]
	In vitro	HepG2	GW4064 (Ac)	↓ p16/INK4a, ↑HNF-4α	[151]
	In vivo	IFNγ-FXR KO mice xenograft	–	↑Liver fibrogenesis, ↑toxic bile accumulation	[152]
	In vitro	HepG2	CDCA (Ac)	↓IL-6, ↑SOCS3, ↓inflammation	[62]
	In vivo	LPS-treated C57BL/6 mice	CDCA (Ac)	↓IL-6, ↑TNF-α, ↑SOCS3, ↓inflammation	[62]
	In vivo	FXR null C57NL/6 N mice	–	↓SHP, ↑CYP7A1,	[153]
Lung Cancer	In vitro	H1975, H1299	Guggulsterone (In)	↓Cell proliferation, ↑G0/G1 phase arrest, ↓cyclin D1, ↓CDK2, ↓CDK4, ↓CDK6, ↓p-Rb	[154]
	In vitro	H1975, H1299	FXR siRNA (In)	↓Cell proliferation, ↑G0/G1 arrest, ↓cyclin D1, ↓p-Rb	[154]
	In vitro	HCC4006	Guggulsterone (In)	↓Cell proliferation	[154]
	In vivo	H1975 xenograft	FXR or NC-shRNA (In)	↓Tumor growth	[154]
Pancreatic Cancer	In vitro	MIA-PaCa2, PANC-1	FXR siRNA (In)	↓Cell proliferation, ↓migration, ↓VEGF, ↓NF-κB DNA-binding	[155]
	In vitro	MIA-PaCa2, PANC-1	Guggulsterone (In)	↓Cell proliferation, ↓migration,↓invasion	[155]
	In vitro	Patient tissues	–	↑FXR, ↑Sp1	[156]
	In vitro	BxPC3, PANC-1	GW4064 (Ac)	↑Cell migration, ↑invasion	[156]
	In vitro	CD18/HPAF	CDCA(Ac), DCA(Ac)	↑MUC4, ↑c-Jun, ↑p-Src	[157]
	In vitro	patient tissues	–	↑FXR, ↓HRG, poor prognosis	[158]
Prostate cancer	In vitro	LNCaP	CDCA(Ac), GW4064(Ac)	↑PTEN, ↓p-Akt, ↓cell proliferation	[159]
	In vitro	LNCaP	CDCA (Ac), GW4064(Ac) ADT (Ac)	↓UGT2B15, ↓UGT2B17, ↓glucuronidation of androgens	[160]
Renal Cancer	In vitro	ACHN cells	–	↓p53, ↓p21/Cip1, ↓miR-21	[161]

Ac- activates FXR

In- inhibits FXR

↑ designates increase/activation

↓ designates decrease/downregulation

^a^Combination with cisplatin

### FXR in breast cancer

Breast cancer represents the most frequent cancer in women with high mortality [9, 16, 162–164]. Numerous studies have suggested the role of FXR in several stages of breast cancer and patient’s survival. The activated level of FXR was associated with more prolonged survival of the patients [105]. Further, FXR was highly expressed and retained in the cytoplasm in most breast carcinoma cases which could be correlated to a minor invasive tumour. It was also associated with longer overall disease-free survival in invasive breast carcinoma patients [165]. However, the expression of FXR in Estrogen receptor-positive (ER^+^) carcinoma might be associated with a poor prognosis [111]. The FXR was positively expressed in ER^+^ MCF-7 cells compared to ER^−^ MDA-MB-231 cells [110]. The expression of FXR also correlates with other proteins such as Ki-67, cyclin D1, and p27 in postmenopausal women, as well as other breast cancer biomarkers such as progesterone receptor (PR), GATA Binding Protein 3 (GATA-3), a coactivator of ER, amplified in breast cancer-1(AIB-1), cytokeratin (CK)-8/18 and mucin 1 (MUC1) [110]. The proliferation of ER^+^ cells was enhanced by the activation of FXR with CDCA treatment, where the inhibition of FXR by siRNA and estrogen inhibitors could reverse the effect [110]. The increased FXR-ER dimer formation induced by CDCA results in the enhanced ability of breast cancer cells to induce metastasis to the bone tissues by increasing the expression of runt-related transcription factor 2 (RUNX2) that allows breast cancer cells to mimic the expression pattern and micro-environment of the bone tissues [104]. Similarly, another bile acid i.e., bile acid salt sodium deoxycholate (BSDC) promotes the migration of MDA-MB-231 cells to the bone tissues by activated nuclear accumulation of FXR, urokinase-type plasminogen activator (uPA), and F-actin [113]. Then the treatment of MDA-MB-231 cells with guggulsterone inhibited migration and induced apoptosis. However, the combination of BSDC and guggulsterone treatment showed a decrease in apoptosis [113].

Contrastingly, studies have also reported the tumour suppressive role of FXR in breast cancer. For example, the activation of FXR by GW4064 inhibits leptin and its target genes induced by cancer-associated fibroblasts (CAFs) while increasing the expression of suppressor of the cytokine signalling 3 (SOCS3) that lead to inhibition of cell growth and invasion [106]. The treatment of GW4064 also reduced tumour growth in mice xenograft models injected with MCF-7 cells alone or co-injected with CAFs [106]. The FXR activated by GW4064 inhibited the migration of breast tumour CAFs by decreasing integrin-linked kinase (ILK), Ras homolog family member A (RhoA)-C, cell division control protein 42 homolog (Cdc42), Ras-related C3 botulinum toxin substrate 1 (Rac1)-3, myosin light chain (MLC) and phosphorylated Akt proteins, where the blocking of FXR by guggulsterone resulted in decreased effect of GW4064 [105]. Also, the treatment of MCF-7, MDA-MB-231, and MDA-MB-468 cells with FXR agonists, CDCA, and GW4064 induce apoptosis [107, 112]. Further, the treatment with GW4064 in breast cancer cells increase apoptosis through nuclear condensation and stimulation of FXR target genes such as SHP, multi-drug resistance-associated protein (MRP)-2 and ileal bile acid-binding protein (IBABP) while repressing MDR3, MRP1, solute carrier family 7 member 5 (SLC7A5), aromatase (CYP19), and growth arrest and DNA damage-inducible (GADD) proteins [112]. Besides, the FXR activated by CDCA and GW4064 decreased the proliferation of tamoxifen-resistant MCF-7TR1 cells where CDCA inhibited human epidermal growth factor receptor 2 (HER2) by preventing the transcriptional binding of NF-κB to HER2 promoter [109]. Moreover, the combined treatment of CDCA and tamoxifen produced a similar effect by inhibiting the EGFR-induced phosphorylation of HER2 and activation of mitogen-activated protein kinase (MAPK) [109]. Furthermore, the walnut oil extracts and its components activated FXR and its associated targets that lead to decreased cell proliferation in MCF-7 cells [108].

### FXR in oesophagal cancer

Oesophagal cancer (EC) is represented as one of the deadly and sixth-most frequent malignancies globally [12, 166]. It is defined as the malignancy of the oesophagus categorized into squamous cell carcinoma (SCC) and adenocarcinoma [167]. The former type represents the malignancy derived from the stratified epithelial lining of the oesophagus while later represents the columnar glandular cells that replace the squamous epithelial cells [167]. The bile acids act as an essential factor for the progression of Barrett’s oesophagus (BE) to oesophagal adenocarcinoma (EAC) [168]. The level of FXR is highly expressed in esophagitis, BE, and EAC compared to normal cells, where its expression was higher in BE-cells than EAC cells [120]. The treatment of BE-cells with guggulsterone increased apoptosis by increasing the expression of caspase 3 [169]. The levels of bile acids induce the expression of FXR and miR- 221 and − 222, which decrease the level of p27Kip1 and caused proteasomal degradation of Caudal-related homologue 2 (CDX-2). The inhibition of miR- 221 and − 222 could increase p27Kip1 and CDX2 in EAC cells and decrease tumour growth in vivo [168]. Additional studies also suggest that the overexpression of FXR in EAC tissues contribute to advanced cancer pathological features such as higher tumour grade, increased tumour size, and nodal metastasis. Therefore, the knockdown of FXR by shRNA diminished the cell viability and tumour growth in vitro and in vivo respectively [130]. Similarly, blocking of FXR by guggulsterone also inhibited EC cells through the induction of caspase-mediated apoptosis and inhibition of COX-2 and matrix metalloproteinase-9 (MMP-9) [130].

### FXR in gastric cancer

Gastric cancer represents one of the fatal malignancies in the world with poor prognosis [170, 171]. The expression of CDX2 and FXR was high and positively expressed in gastric metaplasia compared to gastritis [128]. The gastric metaplasia cells treated with CDCA induce the direct interaction of FXR to SHP, which increased the expression of CDX2 that acts as a tumour suppressor protein. While the elevated CDX2 level could be blocked by inhibiting FXR or knockdown of either FXR or SHP proteins [128]. The excessive levels of bile acids often cause inflammation that could lead to gastro-oesophageal cancer and FXR is known to exhibit gastroprotective effects by regulating gastric damage [131]. Thus, the introduction of the FXR gene to the FXR-deficient ACS cells induced protection against TNF-α-induced cell damage and exerts anti-apoptotic effect through its interaction with keratin 13 (K13) [131]. Further, the defensive behaviour of FXR against inflammation was also confirmed in K13-expressed C57BL/6 mouse model which in the absence of FXR develops gastric ulcers [131].

### FXR in lung cancer

Lung cancer is known to be the most common and lethal cancer globally. It represents two broad categories, i.e., small cell lung carcinoma (SCLC) and non-SCLC (NSCLC) [172, 173]. Studies had also reported the role of FXR in lung cancer; for instance, FXR is highly expressed in NSCLC cells where the knockdown of FXR inhibits cell proliferation in vitro and reduced tumour growth in vivo. The inhibition of FXR via siRNA and guggulsterone induces G0/G1 cell arrest by suppressing cyclin D1 and its associated proteins pRB and other cell cycle regulators viz. cyclin-dependent kinase (CDK)-2, CDK4 and CDK6 [154]. In a cohort study with NSCLC patient samples, the inverse relation of FXR to PD-L1 was observed and the subtype FXR^high^PD-L1^low^ was associated with poor survival outcome [22]. FXR also cause immunosuppression by decreasing the proliferation and function of CD8^+^ T-cells in FXR^high^PD-L1^low^ NSCLC cell line. In vivo studies in the Lewis lung carcinoma model also showed that FXR decreased infiltrating immune cells in FXR^high^PD-L1^low^ subtype [22].

### FXR in pancreatic cancer

Pancreatic cancer is also one of the most lethal malignancies in the world with poor prognosis and is primarily classified into pancreatic endocrine tumour and adenocarcinoma [174–179]. The FXR is highly expressed in pancreatic cancer tissues that result in poor survival and poor prognosis in pancreatic patients [156]. The increased expression of FXR was also related to nodal metastasis [155, 180]. Besides, FXR was related to high specificity protein (Sp)-1 expression that increases proliferation and migration of pancreatic cancer cells [156]. The activation of downstream targets of FXR such as p38-MAPK and PI3K/AKT pathways lead to the activation of phosphorylated Sp-1 and its target proteins which increased cancer progression [156]. The FXR activated by bile acids also increased tumour progression by activating focal adhesion kinase (FAK)/c-Jun, Src, and mucin 4 (MUC4) [157]. The increased FXR expression is also inversely related to histidine-rich glycoprotein (HRG) protein that can be correlated to different stages of carcinogenesis such as nodal metastasis, invasion, large tumour size and is associated with poor survival and poor prognosis in patients [158]. However, one of the studies reported that the increased level of FXR and its binding partner RXR (α, β, and γ) are associated with low stage tumour and better survival in patients [181]. Besides, the downregulation of FXR by siRNA or its inhibition by its natural antagonist, guggulsterone, resulted in the suppression of NF-κB activity and its regulated target vascular endothelial growth factor (VEGF), which lead to decreased cell proliferation, invasion and migration of pancreatic cancer cells. However, the presence of an FXR agonist, GW4064, can reverse the process and increase the activity of FXR that increases the progression of pancreatic cancer [155].

### FXR in renal cancer

Renal cancer is more frequent in men than women, representing a high risk in individuals between the ages of 60 and 70 [182]. It comprises various subtypes of malignancy consisting of different modifications of genes and molecules [182]. Renal cell carcinoma (RCC) represents the major types of renal cancer [183]. Overexpression of FXR was reported in RCC cells, and the high cytoplasmic expression of FXR was shown to be more common in women correlated with high histological grades [184]. The activated expression of FXR increased the proliferation of ACHN cells via suppression of p21/Cip1 and p53 in miR21 dependent behavior while the knockdown of FXR could inhibit the proliferation of renal cancer cells [185]. Further, FXR and LXR are known to regulate the Oct3/4 gene. The FXR activated by GW4064 downregulates the expression of Oct3/4 gene in normal HK-2 cells, but this effect was not observed in ACHN cells. This could be due to the shifting function of FXR from cell differentiation in normal cells to increasing cell proliferation in renal cancer cells [161].

As the expression of FXR varies in different cancers, contrasting to the aforementioned studies, in some cancers, low expression of FXR helps in the development and progression [186]. Therefore, the next part of the review discusses the role of downregulation of FXR in different cancers and its effect on various processes of cancer:

### FXR in biliary tract cancer (BTC)

BTC represents one of the rare but lethal type of cancer consisting of two major cancers, i.e., gall bladder carcinoma (GBC) and cholangiocarcinoma (CCA), revealing different histological and clinical features [187]. The CCA is further divided into two types based on its region such as intrahepatic (iCC) and extrahepatic (eCC) [188]. The normal expression of FXR in bile tract tissue is low, which get further diminished in cancer condition [115]. FXR has an inverse relation to miR-421 that act as an oncogenic factor for BTC. The high expression of miR-421 result in increased cancer hallmarks such as proliferation, clonogenicity, and migration in BTC, however, the inhibition of miR-421 could reverse the condition and induce G0/G1 cell arrest [115]. The loss of FXR in iCC cells and tissue samples was also associated with an advanced tumour stage and poor prognosis [119]. The excess levels of bile acids decreased the level and chemoprotective activity of FXR in the bile duct by inducing high inflammation and interleukin (IL)-6 levels that enhanced the cell proliferation [189]. Another study reported that free bile acids such as cholic acid (CA), deoxycholic acid (DCA), and CDCA increased the expression of FXR which is reversed in the presence of bile acids-glycine conjugates such as GCA, GDCA and GCDCA. The effect of the free bile acid, CDCA in the presence of FXR agonist GW4064 resulted in decreased cell proliferation and tumour growth in vitro and in vivo*,* respectively. GW4064 also inhibited the tumour growth induced by GDCA in a CCA mouse model [118]. Further, a study reported that the activation of FXR by GW4064 and CDCA resulted in apoptosis by sensitizing the cancer cells to cisplatin by inhibiting phosphorylated signal transducer and activator of transcription 3 (pSTAT3) and B-cell lymphoma-extra large (Bcl-xL) expressions through FXR-induced SHP cascades [114]. The co-treatment of the FXR agonists with cisplatin also suppressed tumour growth in vivo through SHP-mediated inhibition of pSTAT3 [114]. Additionally, OCA increased the expression of FXR in mucinous and mixed-type iCCA cells [116]. The treatment of iCCA cells with either OCA or CDCA inhibited the cell proliferation where the OCA treatment also initiate apoptosis and prevent migration of iCCA cells. The effect of OCA in iCCA cells was enhanced in combination with gemcitabine or cisplatin which was also observed to reduce tumour growth in vivo [116]. Further, the reduced expression of FXR promotes invasion of CCA cells where the treatment with OCA decreased the expression of Ki67, PCNA, cyclin D1, and D3, and mitochondrial energy metabolism that results in obstruction of cell proliferation and migration and induction of apoptosis in CCA cells. It also reduced tumour growth in a mouse model [117]. Besides, the GBC patients showed low expression of FXR and inversely high expression of myeloid cell leukaemia sequence 1 (Mcl-1) that were associated with higher tumour progression and poor survival of the patients [190]. Contrastingly, another study reported the positive expression of FXR in GBC patients, where the inhibition of FXR could inhibit the glycine conjugated chenodeoxycholic acid (GCDCA)-induced EMT and metastasis of GBC cells [191].

### FXR in bone cancer

Bone cancer, responsible for less than 1% of total cancer cases, can be divided into different categories, such as osteosarcoma, Ewing’s sarcoma, and chondrosarcoma [192–196]. However, it represents a significant cause of mortality in the world [196]. In a recent study, the low expression of FXR was reported in osteosarcoma cells [103]. However, activation of FXR by GW4064 was reported to increase miR-23b-3p, which lead to the suppression of cyclin G1 and cell proliferation and the inhibition of miR-23b-3p reverses this effect in MG-63 bone cancer cells. Therefore, the treatment with GW4064 increase miR-23b-3p and induce G1 phase cell cycle arrest by modulating the expressions of Bcl-2, Bax, and Caspase-3 that result in cell apoptosis [103]..

### FXR in colon cancer

Colon cancer is the third most prevalent cancers in the world with a reported incidence of 1.8 million cases [163, 197–202]. The mortality of 862,000 cases due to this cancer was reported by the World Health Organization (WHO) in 2018 [202]. The first report of the involvement of FXR in colon carcinogenesis revealed that the expression of FXR mRNA was decreased in colon adenomas and carcinomas [127]. Further, the expression of FXR increases with the extent of differentiation in Caco2 and HT29 colon cancer cells [127]. Another study revealed that the loss of FXR increased the progression of tumour in mice model via Wnt signalling by increasing neutrophils, macrophages and TNF-α which lead to increased cell proliferation and decreased apoptosis. However, the activation of FXR reverse the process by inducing apoptosis [126]. The low or diminished expression of FXR was also detected in HCT-116 and SW480 cells and ulcerative colitis patients (with severe inflammation) [203]. Another study in colitis-induced colon cancer mouse model revealed that FXR and FGF15 and its target FGFR4 were downregulated due to less accumulation of bile acids and decreased bile acid transporters in the ileum which lead to the suppression of FXR signaling [204]. Further, the knockdown of FXR increased the migration of colon cancer cells by inducing the expression of EMT markers such as vimentin, snail, slug, fibronectin, and MMP-9 while suppressing E-cadherin and zonula occludens-1 (ZO-1) [205]. Furthermore, the FXR deficiency in a mouse model was correlated with an increase in inflammation and cancer cell proliferation by increasing the expressions of cyclin D1, β-catenin, c-Myc, and IL-6 [129]. Furthermore, FXR inhibits Wnt/β-catenin pathway by interacting with β-catenin that leads to obstruction of the β-catenin/transcription factor 4 (TCF4) complex [120, 205]. Moreover, the activation of FXR by GW4064 or its overexpression inhibits cell proliferation via suppression of Src (Tyr416)-mediated p-EGFR (Tyr845) and its target p- ERK1/2 which leads to an increase in apoptosis in vitro as well as decreased tumour growth in vivo. However, treatment with guggulsterone reversed the effect and increased cell proliferation [124]. The activated FXR also induce death receptor (DR)5 and the combined effect of TNF-related apoptosis-inducing ligand (TRAIL) and GW4064 result in a synergistic inhibition on colon cancer cell proliferation [76]. Further, the overexpression of FXR inhibits MMP-7-induced cell proliferation and invasion [121].

Besides, a study by Martinez-Becerra P et al. suggested that although the overexpression of FXR activated chemoresistance, it was not required for FXR to be upregulated in colorectal cancer to acquire MDR phenotype. Therefore, the treatment of colon cancer cells with guggulsterone did not alter the MDR genes but increase other FXR targets such as organic solute transporter (OST)-β and organic anion transporting polypeptide 1B3 (OATP1B3) [206]. The miR-192 was reported to decrease FXR and its target molecules, OST-β and OATP1B3 in Huh-7 and Caco-2 cells [207]. Furthermore, cisplatin was reported to induce both FXR-dependent and -independent chemoresistance through the expression of BCRP and MRP2 [208]. Besides, the silenced APC causes the methylation of FXR in C57BL/6 J mice that lead to decreased expression of SHP and IBABP and also increase inflammation and tumour growth by inducing cyclooxygenase-2 (COX-2) and c-Myc [122].

Contrastingly, studies have also reported the absence of FXR expression in undifferentiated SW480 carcinoma cells and SW620 metastatic-derived cells [127]. Another study reported that in colon carcinoma, the expression of FXR was associated with the low stage tumor and better survival outcome compared to FXR-negative carcinomas [125].

### FXR in liver cancer

Liver cancer, ranks the fifth-most common cancer in the world, is associated with poor prognosis and high mortality [209, 210]. One of the most widespread liver cancers is hepatocellular carcinoma (HCC) which arises as a result of chronic liver diseases caused by viruses, use of alcohol, or due to fatty liver disease [211]. Several studies have evaluated the association of FXR in liver cancer. One of the studies reported the downregulated expression of FXR in HCC due to cytokines-induced inflammation or inhibition of activated hepatic nuclear factor 1α (HNF1α) [212]. A similar study confirmed the decreased expression of FXR in human HCC tissues and reduced expression of SHP and BSEP. Moreover, the activation of Wnt/β-catenin signalling was reported to increase the development of HCC in the FXR knockout mouse model [147]. Besides, the FXR deficient mouse with elevated liver injury and tumour progression, showed an increase in fibrosis promoting proteins such as collagen, TNF-α, IL-1β, IL-6, MMPs-(2, − 3 & -9), tissue inhibitor of metalloproteinases 2 (TIMP-2), transforming growth factor (TGF)-β1 and α-smooth muscle actin (Sma-α) [146]. Another study on FXR knockout mouse showed a decreased level of SHP and increased levels of CYP7A1, IL-1β, and β-catenin along with its target protein c-Myc [153]. The FXR knockout mice with deleted interferon-gamma (IFNγ) develop hepatocarcinogenesis by increasing the expression of STAT3 and JNK/c-Jun, but restoration of IFNγ via treatment can reverse this condition by activating p53 and inhibiting STAT3 [152]. Besides, the loss of FXR in obese diabetic mice develops liver cancer through increased cell division cycle 25B (Cdc25b), Cyclin D1, and forkhead box protein M1 (FoxM1) [139]. However, the overexpression of FXR inhibited cell proliferation and induced G0/G1 phase arrest by suppressing the mammalian target of rapamycin (mTOR)/S6 kinase (S6K) pathway. The overexpression of FXR also decreased tumour growth in the mice models [134].

The activated FXR was reported to block the β-Catenin/TCF4 complex and cyclin D1 expression [148]. Moreover, the FXR activated by GW4064 suppressed the expression of fibrosis-related markers like plasminogen activator inhibitor-1 (PAI-1) and Col-α1 in wild type (WT) HCC cells [146]. The activation of FXR also reduced LPS-induced liver inflammation via the upregulation of SOCS3 [62]. Further, the activation of FXR by GW4064 leads to the deactivation of oncogene gankyrin (Gank) and induction of tumour suppressor proteins such as p53, CCAAT/enhancer binding protein (C/EBP)α, and hepatocyte nuclear factor 4α (HNF4α), preventing the development of tumour in a mouse model [133]. Similarly, inhibition of Gank as well as induction of C/EBPβ, HDAC1 proteins and other tumour suppressor proteins, through activation of FXR by CDCA and GW4064, inhibited liver carcinogenesis, while, FXR knockout mouse developed tumour with elevated Gank and decreased C/EBPβ levels [138]. GW4064 also induced G1 cell cycle arrest by inducing p21 and suppressor of cytokine signalling 3 (SOCS3) and reducing phosphorylated STAT3, leading to reduced tumor growth [135]. Further, the activation of FXR by OCA in liver cancer also inhibits cell proliferation in vitro in HepG2 cells [133]. In another study, OCA inhibited cell proliferation, invasion, and migration and induced cell cycle arrest and apoptosis of liver cancer cells by increasing caspase 3 and inhibiting STAT3. It also led to increased SOCS3 and decreased janus kinase-2 (JAK-2), IL-1β, and IL-6 levels [137]. However, the treatment of guggulsterone inhibited the outcome induced by OCA in liver cancer cells [137]. Additionally, the activation of FXR by PX20350 and PX20606 resulted in direct regulation of n-Myc downstream-regulated gene 2 (NDRG2) through its repeat sequence 1 (IR1) binding element that caused inhibition in the proliferation and migration of SK-Hep-1 and SK-GI-18 cells. Further, PX20350 also reduced tumour growth and nodal metastasis in a mouse model [136]. The long term administration of PX20606 resulted in high expression of HRG in mouse plasma. This was also confirmed in healthy human subjects in a phase I clinical study of 7 days with oral administration of PX20606. This suggested that HRG acts as a target for FXR and could be used to detect FXR activation [149]. Similar activation of FXR by CDCA induces the expression of tumor suppressive miR-22 and decreases the level of cyclin A2 that leads to increased G0/G1 cell arrest in Huh7 cells. However, the FXR knockout mice showed a reverse effect by increasing cyclin A2 expression [123]. Furthermore, the upregulation of tumour suppressor miR-122 by activated FXR suppressed the insulin-like growth factor-1 receptor (IGF-1R) and cyclin G1 resulting in decreased cell proliferation in cancer cells and tumour growth in a mouse model [143]. The effect of miR-122 activation could be reversed by knocking down FXR through siRNA [143]. The deficiency of FXR increases cyclin D1, p-STAT3, p-JAK-2 , IL- 1β,  IL-6 and bile acids in FXR-knockout mice. However, the FXR-knockout mice with overexpressed SHP leads to reduced liver tumours [144].

Besides, the FXR agonist INT-767 reduces tumour growth and increases hepatoprotection in a mouse model by reducing the expressions of F4/80, IL-1β, IL-6, TNF-α, cyclin D1, proliferating cell nuclear antigen (PCNA), α-smooth muscle actin (α-Sma), CYP7A1 and collagen, and upregulation of FGF15 and SHP [145]. Furthermore, the overexpression of FXR by lentiviral transfection increases SHP, which causes a decrease in cell proliferation. It was further confirmed with inhibited tumour growth in a nude mouse model [142].

Many studies have reported the selective activation of FXR by the bile acids such as CDCA and other related bile acids [30, 213]. However, the abnormal levels of bile acids are also reported to induce inflammation and liver carcinogenesis. The bile acids normally help in the absorption of cholesterol and lipids [214–217]. The bile acid act as a signalling molecule in the pathways that require the activation of the nuclear receptors including FXR [218]. As bile acids act as surfactants, they can cause damage to hepatocytes, which makes it necessary for its tight regulation of bile acids by various molecules such as FXR [75]. Studies have reported that the mammalian Hippo pathway helps in resizing liver and intestinal regeneration, and its target Yes-associated protein (YAP) is required to be regulated to maintain normal cell proliferation [219]. A study reported by Anakk et al. demonstrated that the excessive level of bile acids in the FXR knockout mice model induced spontaneous liver carcinogenesis and activation of YAP as compared to wild type mice [75]. Further, the FXR knockout mice fed with cholic acid increased the level of bile acids that initiate the development of N-nitrosodiethylamine–induced liver tumour [220]. The inflammatory genes and cell cycle proteins were also upregulated in the FXR knockout aged-mice, however, this effect was not observed in wild type mice [220]. Another study suggested that FXR deficiency initiates liver cancer in mice; however, excess bile acids are essential for the progression of tumor via initiating cyclin D1 and suppressing cell cycle inhibitors [221]. Thus, the bile acids beyond their normal level act as a potential tumour promoter in liver cancer and this effect is more in FXR deficient cells or tissues.

However, the increased level of FXR and its target genes involved in different mechanisms of chemoresistance (MOC) such as MOC-1b (ABCB4), MOC-4 (TCEA2) and MOC-5b (C-C motif chemokine ligand (CCL)14, CCL15 and K13), were reported to protect the hepatocytes from certain genotoxic drugs. The MOC-1b is involved in increased export of drug/toxin, MOC-4 enhance the DNA repair and MOC-5b includes the pro-survival balance [140]. Besides, different outcomes associated with FXR were also reported. One of the studies reported that the decreased level of FXR target, BSEP, in HCC tissues and Huh7 and HepG2 cells was related to modified expression of FXR isomers, FXR-α1, and FXR-α2 [150]. The HCC tissues were associated with increased FXR-α1/FXR-α2, TNF-α, and IL-6 levels [150]. The activation of FXR target, SHP (NR0B2) by FXR agonists was also detected in liver cancer cells [23]. Further, knockdown of FXR in HepG2, Huh-7, and HLE cells resulted in an elevated level of p16/INK4a and inhibited the cell proliferation induced by FXR [151]. The CDCA, GW4064, OCA induces the assembly of actin in Huh-7 and Hep3B cell lines, which further promote TGF-β-induced EMT by increasing N-cadherin and phosphorylated FAK activation. However, the treatment with guggulsterone inhibited the TGF-β-induced EMT in Huh-7 and Hep3B cells [23]. A study has also reported that C-terminal-truncated hepatitis B virus X (HBx-ΔC) could induce cancer stemness and initiate cancer relapse through FXR activation in HCC. However, the treatment of MIHA cells containing HBx-ΔC protein with guggulsterone inhibited cell migration and hepatospheres formation in liver cells [141]. Therefore, further studies could be performed to decipher the exact role of FXR in liver cancer.

### FXR in Prostate Cancer (PCa)

PCa is one of the most widespread malignancy in men worldwide [222–225]. The primary PCa is usually treated with conventional treatment methods such as surgery and radiotherapy [226, 227]. However, the advanced form of this cancer do not respond to the treatment modalities and associated with poor prognosis [226]. Studies have reported the low expression of FXR in PCa tissues [159]. The glucuronidation of androgen acts as a major process of inactivation of androgen in prostate cancer [228]. In addition, the inactive androgen was reported to be a potential inducer of FXR while the active form of androgen induces cell proliferation [229]. Various androgens such as dihydrotestosterone (DHT) and androsterone are converted to inactive glucoronides by the enzymes UDP-glucuronosyltransferase (UGT) 2B15 and UGT2B17 [229]. Thus, the activation of FXR by CDCA and GW4064 reduced UGT2B15 and UGT2B17 and also decreased the androgen glucuronidation [160]. However, in another study, the activation or overexpression of FXR by CDCA and GW4064 leads to inhibition of cell proliferation by increasing phosphatase and tensin homolog (PTEN), while the blocking of FXR by siRNA reversed the effect of the agonists [159]. The LNCaP PCa cells exhibited an increased accumulation of androgen-dependent lipids, which were reduced by the treatment with CDCA. This led to inhibition of SREBP1 and its targets such as fatty acid synthase (FASN), acetyl-CoA Carboxylase (ACC), and ATP Citrate Lyase (ACLY), suggesting the regulation of lipid metabolism by FXR in PCa which decreased cell proliferation in LNCaP cells [230].

### FXR in other cancers

In addition to the role of FXR in the aforementioned cancers, shreds of evidence also proved its role in other cancers viz. such as Leydig cell tumours (LCTs) and thyroid cancer [22, 231, 232]. One of the studies suggested that the presence of the expression of FXR was more in R2C LCTs cells as compared to normal testicular cells [231]. However, the FXR activated by GW4064 and CDCA in R2C cells binds to steroidogenic factor 1 (SF-1) response site in the promoter region of aromatase and inhibits its activity thereby blocking estrogen signalling and reducing tumour cell growth [231]. Besides, the activation of FXR by GW4064 caused an inhibition of cell proliferation and induction of apoptosis in R2C cells by initiating poly (ADP-ribose) polymerase (PARP) cleavage and DNA fragmentation through elevated p53 and p21(WAF1/Cip1) and binding of FXR/NF-κB within the promoter region of p53 [132]. Similarly, the treatment of GW4064 in an LCT mouse model reduced tumour growth [132]. Besides, a high level of FXR expression was also observed in thyroid neoplasia and the expression was more common in papillary thyroid carcinomas, which is associated with higher lymph node metastasis and invasion, and high recurrence rate [232]. Thus, these studies suggest the diverse expression of FXR in different cancers and care should be taken while targeting FXR.

FXR is differentially expressed in different cancers and accordingly it modulates the development of these cancers. Therefore, the agonists and antagonists of FXR have differential roles in various cancers. Hence, the next part of the review discusses the important agonists and antagonists of FXR.

## Agonists of FXR

The bile acids act as an agonist for FXR to perform its various functions [233]. The bile acid increases the progression of premalignant state to carcinoma in EC [168]. The bile acid CDCA, could induce the activation of FXR and cause the proliferation, migration, and metastasis of breast cancer cells [104, 110]. Besides, BSDC, which is another bile acid, induced the activation of FXR that results in increased migration of the MDA-MB-231 cells [113]. The treatment of GW4064, another agonist of FXR increases cell proliferation, invasion and migration by activating NF-κB and VEGF in pancreatic cancer cells [155]. Additionally, CDCA, GW4064, and OCA promote the migration of HCC cells by inducing N-cadherin [23].

Interestingly, some of the studies also reported the anticancer activities of FXR agonists. For instance, CDCA was reported to inhibit cell proliferation in tamoxifen-resistant breast cancer cells by suppressing the expression of HER2 [109]. The treatment of an agonist, OCA, was also reported to inhibit proliferation and migration and induce apoptosis in CCA cells where this outcome was more prominent with its combination with gemcitabine or cisplatin [116]. Another agonist, GW4064, was also reported to inhibit cell proliferation in various cancers such as liver cancer, LCTs, and colon cancer [76, 132, 135]. Further, GW4064 induces apoptosis in breast cancer cells by regulating the activation of FXR and by modulating the FXR-associated targets such as SHP, MRP-2, IBABP, MDR proteins, solute carriers, aromatase, and GADD [112]. Further, the agonists of FXR, i.e., PX20350 and PX20606, inhibited the proliferation and migration of SK-Hep-1 and SK-GI-18 liver cancer cells [136].

## Antagonists of FXR

One of the main antagonist of FXR, i.e., guggulsterone, induced apoptosis in EC cells by increasing the expression of caspases [130]. The treatment of breast cancer cells with guggulsterone inhibited migration and induced apoptosis by inhibiting FXR, uPA, and F-actin [113]. Guggulsterone was also reported to inhibit EMT markers, VEGF, and NF-κB in HCC and pancreatic cancer [23, 155]. Moreover, the inhibition of FXR by siRNA inhibited cancer cell proliferation in ER-positive breast cancer and NSCLC cells [110, 154]. Further, miR-192 inhibited the activity of FXR and its targets OST-β and OATP1B3 in colon cancer [207]. Contrastingly, the treatment of colon cancer cells with guggulsterone inhibited FXR and induced proliferation [124]. Thus, the antagonists perform their activities depending on the expression of FXR in different cancers.

## Conclusion and future perspectives

Cancer is one of the leading causes of mortality associated with multiple modifications in different molecules and cell signalling pathways that perform a diverse range of normal function and metabolism [234–237]. FXR is a nuclear receptor that is usually involved in regulating the levels of bile salts, cholesterol, lipids and glucose metabolism. The imbalances in these functions result in the development of several diseases. For instance, FXR is also involved in regulating cancer by modulating a range of molecules associated with the cancer signalling pathways. Studies had suggested the tissue-specific and cancer-specific function of FXR. These studies also indicated that the elevated level of FXR was associated positively with a high rate of tumour progression in breast, lung, and pancreatic cancers [154, 165, 180]. While in other cancers such as CCA and liver cancer, the low expression of FXR increased the cancer progression and were also associated with poor prognosis [115, 212]. Besides, the expression of FXR varies in different types of cells of the same cancer. For example, FXR is highly expressed in ER-positive MCF-7 cells compared to ER-negative MDAMB-231 cells [110]. The expression of FXR also varies between differentiated and undifferentiated colon cancer cells [127]. Due to the differential expression, the agonists and antagonists of FXR perform their activities differently. Further, FXR is involved in regulating a wide range of molecules such as tumour suppressor proteins p53, p-Rb, and C/EBPβ; cell cycle regulators such as cyclins, CDKs and CDK inhibitors; cytokines such as IL-(1β and 6), TNF-α and transcription factors such as NF-κB and STAT3; proteins involved in differentiation, RUNX2 and Oct3/4; EMT and angiogenic markers, and chemoresistance proteins. FXR also modulates several signalling pathways such as EGFR/ERK, NF-κB, TGF-β, p38/MAPK, PI3K/AKT, and Jak/STAT that lead to the regulation of various cancers. Thus, considering the diverse expression and role of FXR, it could be regarded as a potential target in the treatment of different cancers. Further, as various cancers have differential expression, i.e., overexpression and low expression of FXR, it should be targeted carefully and more studies should be performed to understand the long-term effects of agonists and antagonists. As FXR is differentially expressed in various cancers, the therapies should be design in such a way that the drug reaches only to the particular organ or tissues. Besides, the role of FXR in chemosensitization and radiosensitization should be studied in-depth, which would help us target this protein for the better management of this disease.

## Data Availability

Not Applicable.

## Abbreviations

ACR: Acyclic retinoid
ADT: Androsterone
AOM: Azoxymethane
BA: Bile acids
Bcl-xL: B-cell lymphoma-extra large
BSDC: Bile acid salt sodium deoxycholate
BSEP: Bile salt export pump
BSP: Bone sialoprotein
BTC: Biliary tract cancer
CAC: Colitis-associated cancer
CAF: Cancer-associated fibroblast
CCA: Cholangiocarcinoma
Cdc42: Cell division control protein 42 homolog
CDCA: Chenodeoxycholate
Cdk4: Cyclin-dependent kinase4
C/EBPα: CCAAT-enhancer-binding protein alpha
CCNG1: Cyclin G1
c-Myc: Cellular myelocytomatosis
CPT-I: Carnitine palmitoyltransferase I
CYP7A1: Cholesterol 7α-hydroxylase
Cyt-c: Cytochrome c
DCA: Deoxycholic acid
DR5: Death receptor 5
EAC: Esophageal adenocarcinoma
EMT: Epithelial mesenchymal transition
ER: Estrogen receptor
ERK: Extracellular signal-regulated kinase
FXR: Farnesoid X receptor
FXR-DN: Dominant negative FXR protein
GADD: Growth arrest and DNA damage–inducible protein
Gank: Gankyrin
HC: Hepatocarcinogenesis
HCC: Hepatocellular carcinoma
HDCA1: Histone deacetylase 1
HER: Human epidermal growth factor receptor
HNF-4α: Hepatocyte nuclear factor 4 alpha
hPPARα: Human peroxisome proliferator-activated receptor α
HRG: Histidine-rich glycoprotein
IBABP: Ileal bile acid-binding protein
iCC: Intrahepatic cholangiocarcinoma
IL: Interleukin
ILK: Integrin-linked kinase
JAK: Janus Kinase
JNK: c-Jun N-terminal kinase
KO: Knockout
LCA: Lithocholic acid
LCT: Leydig cell tumors
MCF-7 TR1: Tamoxifen-resistant breast cancer cells
MDR: Multidrug resistance protein
MIP3α: Macrophage inflammatory protein 3α
MLC: Myosin light chain
MRP2: Multidrug resistance-associated protein 2
mTOR: Mammalian target of rapamycin
MUC4: Mucin 4
NF-κB: Nuclear factor-kappa B
OC: Osteocalcin
OCA: Obeticholic acid
OPN: Osteopontin
PAI-1: Plasminogen activator inhibitor-1
PCNA: Proliferating cell nuclear antigen
PR: Progesterone receptor
PMA: Phorbol-12-myristate-13-acetate
PO: Peppermint oil
PTEN: Phosphatase and tensin homolog
RhoA-C: Rho and actin cytoskeleton
ROS: Reactive oxygen species
RXR: Retinoid X receptor
RUNX2: Runt-related transcription factor
S6K: S6 kinase
SCFA: Short chain fatty acids
SHP: Small heterodimer partner
SLC7A5: Solute carrier family 7 member 5
SOCS3: Suppressor of cytokine signaling 3
SOD3: Superoxide dismutase 3
Sp1: Specificity protein
STAT3: Signal transducer and activator of transcription 3
TCA: Taurocholate
UGT: UDP-glucuronosyltransferase
uPA: Urokinase-type plasminogen activator
uPAR: urokinase-type plasminogen activator receptor
